# The role of working memory in the temporal control of discrete and continuous movements

**DOI:** 10.1007/s00221-014-4108-5

**Published:** 2014-10-14

**Authors:** Pieter-Jan Maes, Marcelo M. Wanderley, Caroline Palmer

**Affiliations:** 1 Department of Psychology, McGill University, 1205 Dr Penfield Ave, Montreal, QC H3A 1B1 Canada; 2 Department of Music Research, CIRMMT, McGill University, Montreal, QC Canada

**Keywords:** Timing, Working memory, Discrete movements, Continuous movements, Music performance

## Abstract

Music performance requires precise control of limb movements in order to achieve temporal precision of performed tone onsets. Previous findings suggest that processes recruited for the temporal control of rhythmic body movements, such as those required in music performance, depend on the movement type (discrete vs. continuous) and the rate of the produced interonset intervals (sub-second vs. supra-second). Using a dual-task paradigm, the current study addressed these factors in the temporal control of cellists’ bowing movements. Cellists performed melodies in a synchronization-continuation timing task at a specified fast (intertone interval = 700 ms) or slow (intertone interval = 1,100 ms) tempo with either discrete (staccato) or continuous (legato) bowing movements. A secondary working memory task involved a concurrent digit-switch counting task. Analyses of the produced tone durations showed that the working memory load significantly impaired temporal regularity when the melodies were performed with discrete bowing movements at the slower tempo. In addition, discrete movements led to more errors on the working memory task. These findings suggest that continuous body movements provide temporal control information to performers under high cognitive load conditions.

## Introduction

Many everyday activities, such as walking, dance, and some sports, require precise temporal control of body movements that are rhythmic: they exhibit quasiperiodic timing (Hogan and Sternad 2007). Research on motor control and coordination suggests that the timing of rhythmic body movements is a hybrid phenomenon (Zelaznik et al. 2008), in the sense that different control processes regulate the timing of rhythmic movements depending on the type of movement used. Typically, a distinction is made between *discrete* and *continuous* rhythmic movements (Delignières et al. 2004; LaRue 2005; Robertson et al. 1999; Studenka et al. 2012; Torre and Balasubramaniam 2009; Zelaznik et al. 2002). Discrete rhythmic movements are characterized by salient events that are separated by pauses (intervals during which there is no movement). In contrast, continuous rhythmic movements are continuous and smooth and do not include interspersed pauses. A second distinction in the temporal control of rhythmic movements is one between sub-second intervals (interonset interval, IOI < 1 s) and supra-second intervals (IOI > 1 s), corresponding, respectively, to fast and slow rates or tempi (Buhusi and Meck 2005; Lewis and Miall 2003).

The control processes underlying temporal coordination have been attributed to a wide range of brain areas, including the cerebellum, the basal ganglia, neocortical areas, and prefrontal areas (Diedrichsen et al. 2003; Lewis and Miall 2003). Timing of discrete movements is posited to be controlled by *event timing,* related to the metaphor of a clock or stopwatch, and is described as *timing*-*with*-*a*-*timer* (Zelaznik et al. 2008). The basic idea is that timing is explicitly controlled by a dedicated internal clock that is capable of metering out time. The most influential account of event timing is the pacemaker-accumulator model (Gibbon 1977). In this model, a clock (*pacemaker*) emits pulses that enter an *accumulator* via an attention-controlled switch. During the timed interval, collected pulses are stored in working memory and compared with the criterion interval in reference memory. Several studies show that a working memory load interferes with the production of regularly timed (supra-second) intervals (Brown 1997; Fortin and Breton 1995; Krampe et al. 2010; Ogden et al. 2011; Rattat 2010). Additional research has suggested a role of the cerebellum in event timing (Ivry and Keele 1989; Spencer et al. 2007). In contrast, timing by means of continuous body movements recruits an alternative, *emergent* timing system (Zelaznik et al. 2008). In this view, temporal regularity is a property that emerges from the control of movement dynamics, rather than from an explicit, internal clock. The emergent timing account attributes the temporal control of body movements to a dynamical coupling of action and perception, whereas the event timing account assumes the temporal control arises from dedicated internal clock mechanisms.

Additional research indicates that different neural control processes are recruited depending on the duration of the IOIs being timed. The timing of brief, sub-second intervals (IOI < 1 s) is thought to be controlled by an automatic system employing motor planning regions such as bilateral supplementary motor area (SMA) and the left sensorimotor cortex, while the timing of larger, supra-second intervals (IOI > 1 s) is thought to be controlled consciously with effort and recruits prefrontal and parietal regions (Buhusi and Meck 2005; Lewis and Miall 2003).

The current study investigates how continuous and discrete body movements support the production of regular short and long durations in the performance of a naturalistic task, cello performance that normally recruits all types of movement and includes both sub-second and supra-second intervals between tones. In contrast to former studies that typically rely on finger tapping and circle- or line-drawing (Bangert et al. 2011; Bo et al. 2008; Delignières et al. 2004; Elliott et al. 2009; Howard et al. 2011; Huys et al. 2008; LaRue 2005; Lorås et al. 2012; Robertson et al. 1999; Spencer et al. 2003; Studenka et al. 2012; Zelaznik et al. 2002, 2005), the use of discrete and continuous movements is equally familiar to experienced cellists. Cellists use bowing strokes that are both continuous and discrete on the surface of the strings, to produce smooth continuous sound or abrupt discrete sounds, respectively. To our knowledge, no studies have been conducted with production tasks like cello performance that naturally incorporate both movement types (discrete vs. continuous) and manipulations of tone interonset intervals, IOIs (sub-second vs. supra-second). Thus, music performance provides an excellent naturalistic context in which timing can be studied in relation to both movement type (cf. articulation, staccato vs. legato) and tone IOIs.

We applied a dual-task paradigm to investigate the role of working memory in the temporal control of cellists’ bowing movements. The dual-task paradigm assumes that interference occurs when two tasks tap into the same cognitive and sensorimotor resources. Hence, the study of interference patterns can lead to a better understanding of the control and processing systems underlying the execution of temporal production of musical tones in which we vary the movement type and tone IOIs. The primary task was a typical synchronization-continuation timing task (Bravi et al. 2014; Grondin 2003) in which cellists performed simple tone sequences as regularly (evenly) as possible at a specific target tempo. Cellists performed these tone sequences with both discrete bowing movements (i.e., staccato articulation), and with continuous bowing movements (i.e., legato articulation). The target tempo at which they performed—as indicated by a metronome—was either fast (IOI = 700 ms), or slow (IOI = 1,100 ms). Furthermore, two different tone sequences, termed congruent and incongruent, were created based on the relationship between pitch change and bow movement direction (described below). The incongruent tone sequence was expected to demand more cognitive resources, in addition to those required for performing the secondary working memory task. Consequently, we expected that the working memory task would interfere more with the timing task for incongruent tone sequences than for congruent tone sequences. Analyses of the primary task of cello performance focused on mean tone IOIs and the consistency of tone onsets (coefficients of variation) in relation to the target tempo.

The main aim of the experiment was to investigate how the tempo (measured by mean tone IOIs) and the consistency of cellists’ production timing suffered from an additional cognitive load. Therefore, we added a secondary working memory task that cellists were asked to perform concurrently with the primary timing task. We used a digit-switch task that required participants to maintain and manipulate digit information in working memory (cf. Krampe et al. 2010). We hypothesized that an additional cognitive load would affect the mean tempo and the temporal consistency differently depending on the specific movement type. The working memory task was expected to disrupt the temporal control of discrete bowing movements more than continuous bowing movements, because discrete movements included pauses that create distinct events which in turn require internal clock resources to keep track of time. Correspondingly, the working memory task should create greater disruption of timing at slower production rates (IOI = 1,100 ms, supra-second) than at faster rates (IOI = 700 ms, sub-second). Additionally, cellists were expected to exhibit more errors on the working memory task when playing staccato than when playing legato and when playing at slower tempi than when playing at faster tempi. Finally, we hypothesized that the performance of the incongruent sequence, which demands more cognitive resources, would be more affected than the congruent sequence by the working memory task.

## Methods

### Participants

Sixteen cellists (9 females, mean age = 23.8 years; range = 20–33 years), recruited from the Montreal area music community, participated in the study. All were experienced musicians with an average of 13.7 years of formal cello instruction (range = 7–20 years), and average practice of 21.6 h per week (range = 3–35 h). All reported to be right-handed and to not have any hearing problems. Participants received $25 in compensation for the experiment. Written informed consent was obtained prior to participation, and the Ethical Review Committee of McGill University reviewed the experiment.

### Stimulus materials

**Fig. 1:**
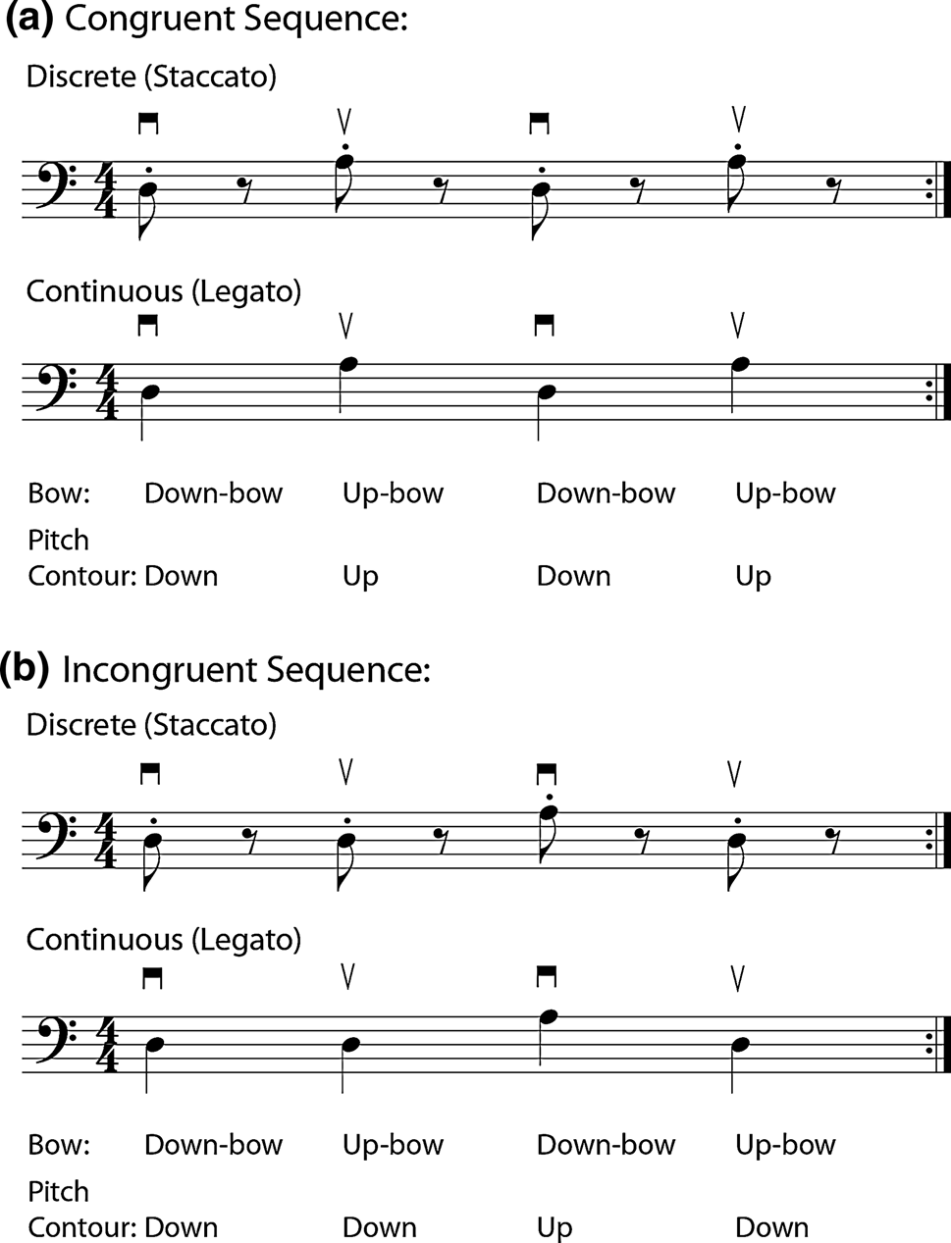
Congruent sequence (**a**) and incongruent sequence (**b**) performed by cellists

Two novel melodies (tone sequences) were created for the primary timing task, each consisting of a four-note repeating pattern (see Fig. 1). The pitches used were D3 (146.8 Hz) and A3 (220 Hz), to be bowed on open strings (i.e., with no fingers placed on strings) to reduce movement requirements. The DADA pattern of the first congruent sequence was chosen to establish an association between cellists’ bow direction (up/down) and pitch class (D/A), whereas the DDAD pattern of the second incongruent sequence established a disassociation between bow direction and pitch class. The D3 and A3 pitches correspond to the tuning of the two thinnest strings of the cello, which require less tension to become activated; these strings were chosen to reduce the task difficulty. Metronome ticks, sounded at the beginning of each trial, indicated the tempo at which the tones should be played; these were produced by a Dr. Beat metronome and were sounded over Dynaudio BM5A MKII speakers at a comfortable listening level. The melodies were designed and notated to be repeated without pauses; performers were instructed to repeat the melodies without stopping on each trial, after which a bell sounded to indicate the end of the trial.

Digit strings of 18 digits length were created for the secondary working memory task, composed of single-digit numerals (1–9) ordered randomly on each trial, excluding immediate repetitions of the same single digit. The digit strings were presented on a computer screen placed in front of the participant about 1.5 m away. Each trial in the working memory condition presented one of the digits every 1,700 ms for the duration of the continuation phase of a trial; this spacing ensured that the digit presentation did not align with tone onsets in either tempo condition.

### Equipment

The experiment was held in a sound-attenuated room of size 4.85 × 5.50 × 3.40 m. Participants sat on a chair whose height could be altered for the comfort of the participant, positioned in the center of the room. The participants were asked to bring their own cello to perform. Movements of the participants, their bow, and cello were recorded using the Qualisys Oqus 400 12 camera Motion Capture System, and corresponding QTM software (http://www.qualisys.com/). The system allowed capture of the three-dimensional position of small (3 mm) spherical infrared (IR) reflective markers at a sampling rate of 210 Hz. The position of the cameras was placed in a circle-like formation around the cellist’s seat. Ten markers were attached to the cello and to the limbs of the performers: one centered just below the fine tuners onto the tailpiece, one centered on the scroll, one on the front-right edge of the cello’s body at the height of the bridge, and one on the top of the cello’s body just next to the fingerboard, the bow (balance point, and tip), and the hand and arm of the cellists (right hand, right elbow, right shoulder, and forehead.

### Design and procedure

The experimental design included 2 tempi (fast/slow) × 2 articulation (legato/staccato) × 2 task (single task/dual task) × 2 melodies (congruent/incongruent) within-subjects repeated measures design, resulting in 16 conditions. Conditions were grouped in pairs of blocks, with each pair representing the single-task and dual-task version of the same tempo/articulation combination, and the single task always preceded the dual-task version. The order of the tempo conditions, the articulation conditions, and the sequences used within each block were randomized. Each trial within each condition was repeated three times. This design yielded a total of 16 × 3 = 48 trials.

Upon arrival, each cellist was informed about the procedure and signed a consent form. Then, they completed a questionnaire assessing their basic demographics, musical background, handedness, and any hearing problems. Afterward, the markers used for capturing movement were attached to the cello, the bow, and the hand and arm of the participant. Next, the cellist performed the primary timing task on practice trials. Each trial presented a synchronization-continuation task (Wing and Kristofferson 1973a) in which a metronome marked the beginning of the synchronization phase and produced equally spaced ticks with an IOI of either 700 ms (fast tempo) or 1,100 ms (slow tempo). The participant was asked to listen to the first two ticks and to start playing one of the two melodies from the third tick onwards, aligning each tone onset—either with staccato or legato articulation—with each tick. Participants were instructed to continue to perform the musical sequence either with staccato or legato articulation: They were told that tones in the staccato articulation condition need to be very short and detached from each other and that tones in the legato articulation condition need to be long and continuous (with no gaps). Participants were instructed to try to keep the bow on the strings and to keep other body parts (feet, head, etc.) as still as possible while playing. They were also informed that the timing of the tones, rather than the sound quality, was of most importance. The metronome ended after ten ticks and the continuation phase commenced, during which participants were asked to repeat the melody while continuing at the tempo indicated by the initial metronome until they heard a bell tone indicating the end of a trial. Each trial required the cellist to repeat the melody 15 times at the fast tempo (for IOI = 700 ms) or 11 times at the slow tempo (for IOI = 1,100 ms) without stopping. Participants practiced the primary task until they felt comfortable with it.

Then participants practiced the secondary task. On each trial, 18 single digits were displayed in random order on a computer screen; each digit was displayed for 1,700 ms and was immediately followed by another digit. The participant’s task was to count silently the number of switches from odd to even numbers, and from even to odd numbers. After the presentation of the series of 18 digits, participants were asked to report to the experimenter the two values representing the number of switches from odd to even numbers, and from even to odd numbers. After each trial, participants were given feedback about the correct number of switches. Participants were presented with practice trials until they had given correct answers on two successive trials.

Finally, participants practiced combining the primary and secondary tasks. Each trial began with the synchronization task for the primary timing task, and the working memory task commenced in the continuation phase after a time span corresponding to eight target tones to allow for tempo stabilization, as in Krampe et al. (2010). After the presentation of the last digit, the bell tone sounded to signal the end of the trial, three target IOIs later, and participants reported the two values representing the number of switches from odd to even numbers, and from even to odd numbers, until they gave correct answers on two successive trials.

In the test phase of the experiment, participants performed the primary task with and without the secondary task in all tempo, articulation, and sequence conditions. Before each trial, participants were informed about which sequence to play, how to play it (i.e., articulation), the tempo, and whether or not they had to perform the additional working memory task. When participants were ready, the experimenter started the metronome to commence a trial. Between blocks, participants had a short break. After each experimental trial that included the working memory task, participants were given feedback about their answers. The entire experiment lasted approximately 2 h.

### Data analysis

**Fig. 2:**
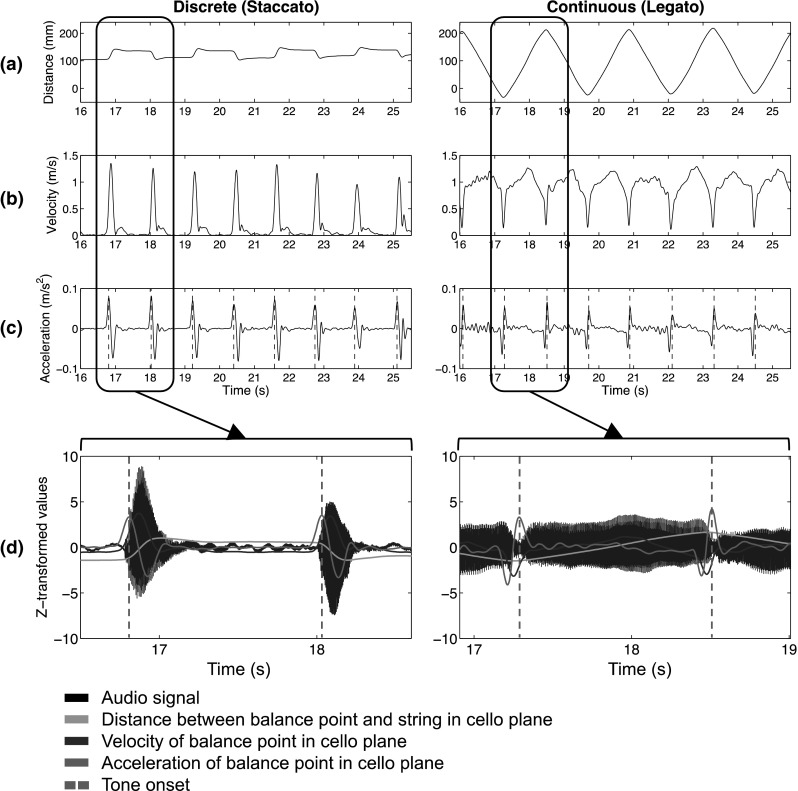
**a** Distance of the bow’s balance point from the string (*left* discrete bowing movements, *right* continuous bowing movements). **b** Velocity of the bow’s balance point. **c** Acceleration of the bow’s balance point (with peaks indicating tone onsets). **d** Audio waveform, with corresponding bow distance, velocity, and acceleration, with peaks indicating tone onsets, for tone onsets in same trial of the congruent sequence (target IOI = 1,100 ms). Values are represented as z-scores in order to increase visibility

Cellists’ tone onsets were determined from the motion of the bow’s balance point relative to the cello’s strings. Because cellists received the explicit instruction to keep their bow on the strings while playing the melodies, movement of the bow’s balance point parallel to the frontal plane of the cello’s body and perpendicular to the strings resulted in sound production. A typical profile of the bow movement (position of the bow’s balance point relative to the strings) in this plane is displayed in Fig. 2a. The first (Fig. 2b) and second (Fig. 2c) derivative of the position values corresponds to the velocity and acceleration of the bow’s balance point movement, respectively. The acceleration values in Fig. 2c/d show distinct peaks in the signal which aligned with acoustic tone onsets; therefore, tone IOIs were computed from the acceleration values (based on local maxima). The mean tone IOIs per trial and the coefficient of variation per trial (SD/mean IOI) (as a measure of temporal consistency) were then computed. Outlier values (exceeding 2SD from trial means), occasionally arising from poorly detected onsets, were excluded from further analysis (IOI = 4.84 %; CV = 2.73 %). Correct answers in the working memory task were determined as the proportion of correct answers per condition, based on participants’ reports of the number of switches between odd and even numbers during the trial. Responses for both the number of odd to even switches and even to odd switches had to be answered correctly for the trial to be considered correct. Four-way repeated-measure ANOVAs were applied to both performance timing (primary task) and memory recall (secondary task) measures with tempo (fast/slow), articulation (discrete/continuous), task (single/dual task) and sequence (congruent/incongruent) as within-subject factors.

## Results

Cellists’ mean proportion correct responses in the secondary working memory task showed significant differences only across articulation conditions, *F*(1,15) = 6.28, *p* = 0.024, indicating higher performance during continuous bowing movements (*M* = 0.73, SEM = 0.048) than discrete bowing movements (*M* = 0.61, SEM = 0.069). Only trials on which the secondary memory task was performed correctly were included in analyses of primary cello performance task.

### Mean cello tone IOIs

As expected, the mean-produced IOIs changed across tempo conditions; mean IOI = 692.6 in the 700 ms condition, mean IOI = 1,073.8 in the 1,100 ms condition; separate analyses were therefore conducted for each tempo condition. The 700 ms condition yielded significant main effects of articulation, *F*(1,15) = 16.84, *p* = 0.001, task, *F*(1,15) = 19.20, *p* = 0.001, and sequence, *F*(1,15) = 5.77, *p* = 0.030. No significant interaction effects were found. Discrete bowing movements were significantly faster (*M* = 682.4, SEM = 5.17) than continuous bowing movements (*M* = 702.7, SEM = 3.42), and cellists played faster on average during the dual task (*M* = 687.9, SEM = 3.55) than the single task (*M* = 697.2, SEM = 3.96). On average, the incongruent sequence (*M* = 688.60, SEM = 4.48) was played faster than the congruent sequence (*M* = 696.51, SEM = 3.38). The same analysis of the 1,100 ms condition indicated significant main effects of articulation, *F*(1,15) = 6.29, *p* = 0.024, and Task, *F*(1,15) = 19.51, *p* < 0.001. As in the faster tempo, cellists performed the discrete movement condition faster (*M* = 1,061.26, SEM = 11.63) than the continuous movement condition (*M* = 1,086.38, SEM = 6.37), and they performed faster during the dual task (*M* = 1,064.00, SEM = 7.73) than the single-task conditions (*M* = 1,083.63, SEM = 8.71). Again, no significant interaction effects were found.

### Coefficients of variation

**Fig. 3:**
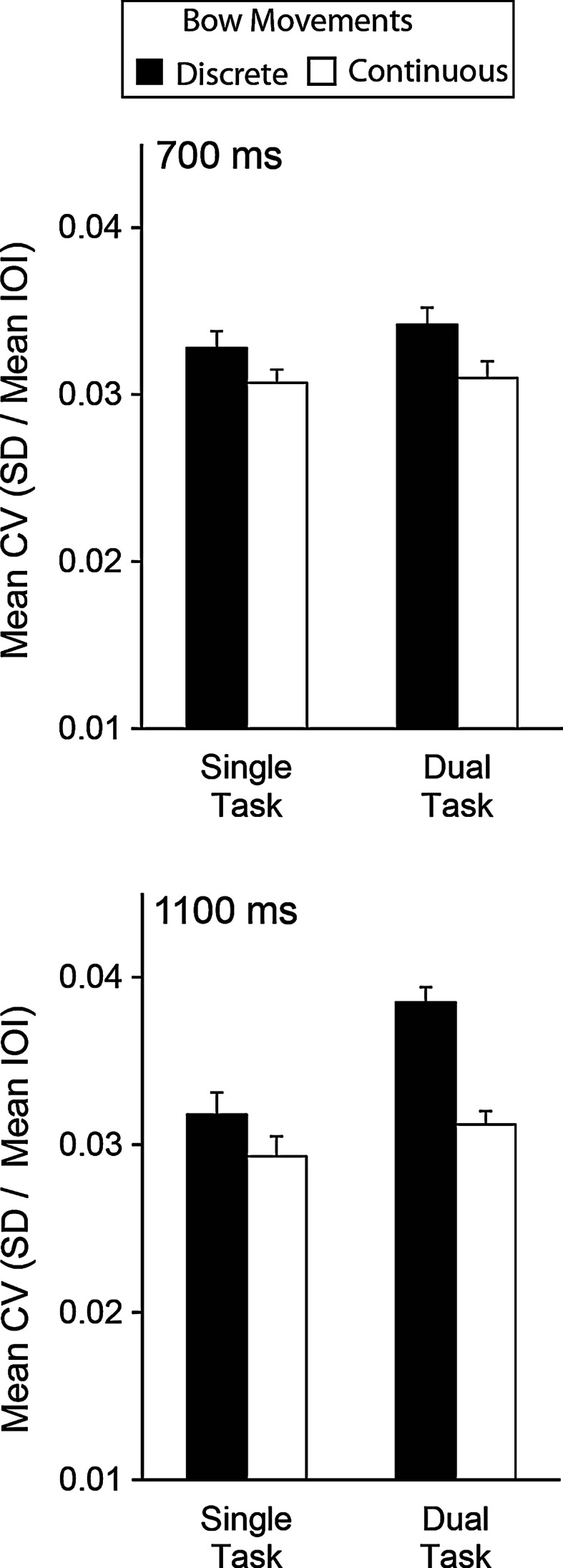
Mean coefficients of variation (with SE bars) by tempo (*top* 700 ms, *bottom* 1,100 ms), task (single/dual), and movement type (discrete/continuous)

Figure 3 shows the mean CV values by condition. The cellists exhibited higher variability during the dual-task condition (*M* = 0.0337, SEM = 0.00061) than during the single-task condition (*M* = 0.0312, SEM = 0.00059), *F*(1,15) = 11.76, *p* = 0.004. There was also higher variability during the discrete movement condition (*M* = 0.0343, SEM = 0.00078) than during the continuous movement condition (*M* = 0.0306, SEM = 0.00066), *F*(1,15) = 11.80, *p* = 0.004. Finally, the CV values for the incongruent sequence (*M* = 0.0336, SEM = 0.00061) were greater than for the congruent sequence (*M* = 0.0313, SEM = 0.00049), *F*(1,15) = 16.01, *p* = 0.001.

The hypothesis that the secondary memory task would affect discrete bowing movements more than continuous bowing movements was evidenced in the significant interaction between articulation and task, *F*(1,15) = 10.94, *p* = 0.005. As shown in Fig. 3, variability increased in the dual task when cellists performed with discrete bowing movements, but not when they performed with continuous bowing movements. The mean CV for discrete bowing movements in the dual-task condition (*M* = 0.0363, SEM = 0.00077) was significantly higher than that of the single-task condition (*M* = 0.0323, SEM = 0.00096), *t*(15) = 5.19, *p* < 0.001; differences across continuous movement conditions did not reach significance.

**Fig. 4:**
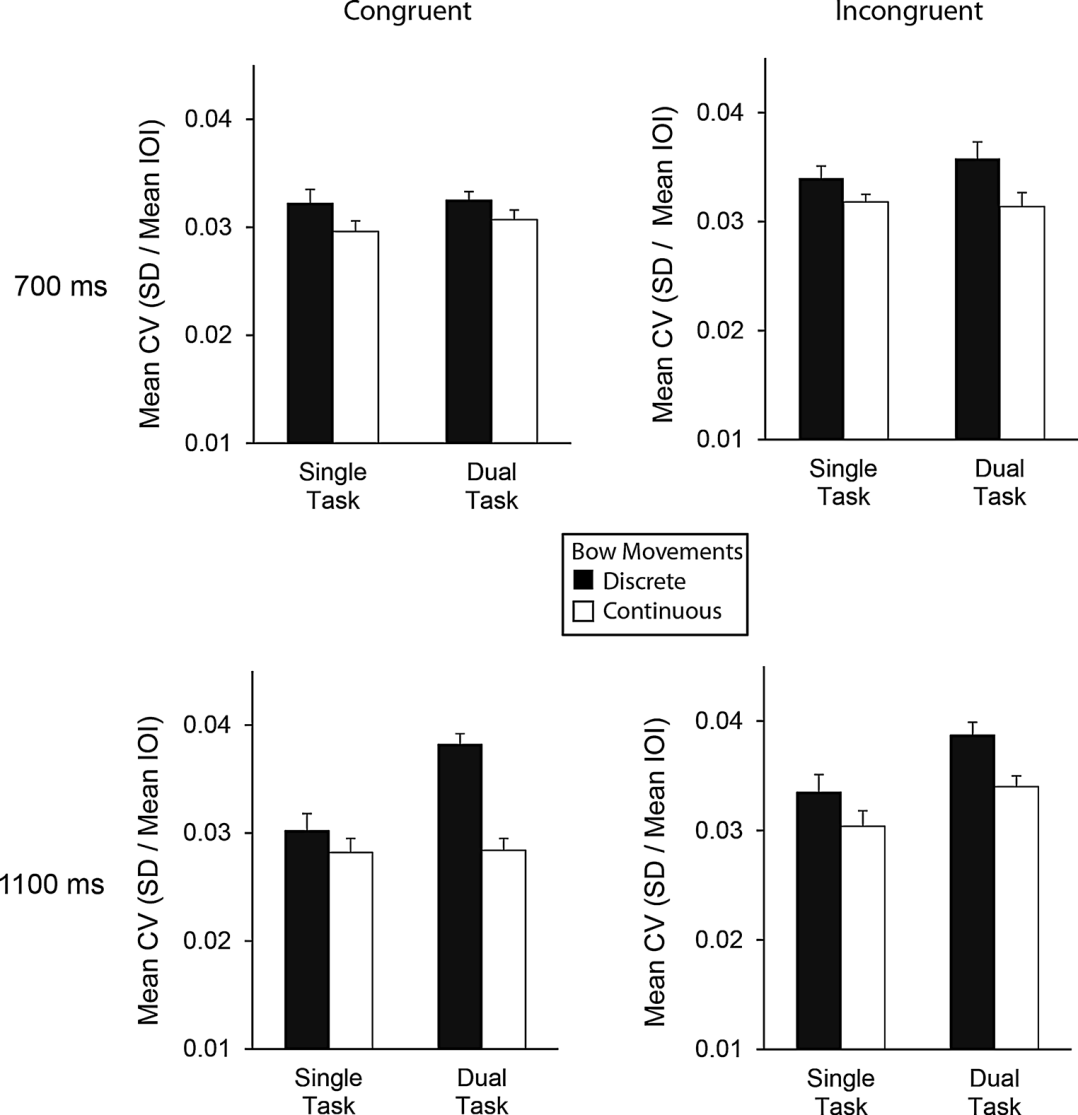
Mean coefficients of variation (with SE bars) by tempo (*top* 700 ms, *bottom* 1,100 ms), task (single/dual), movement type (discrete/continuous), and sequence (*left* congruent, *right* incongruent)

The second hypothesis that the secondary memory task would affect bowing movements produced at longer IOIs more than at shorter IOIs was evidenced in the significant interaction between tempo and task, *F*(1,15) = 13.83, *p* = 0.002. As shown in Fig. 4, the mean CV values in the 1,100 ms condition were greater in the dual-task condition (*M* = 0.0348, SEM = 0.00071) than in the single-task condition (*M* = 0.0306, SEM = 0.00070), *t*(15) = 6.24, *p* < 0.001; the same comparison did not yield significant differences for the 700 ms tempo condition, *t*(15) = 0.83, *p* = 0.42.

In addition, the two hypotheses: that working memory most affects discrete movements and slower tempi, combined to yield decreased consistency in cellists’ bowing. A significant three-way interaction between articulation × tempo × task, *F*(1,15) = 7.09, *p* = 0.018 indicated lowest timing consistency in the dual-task condition with discrete bowing movements in the 1,100 ms condition, *t*(15) = 5.01, *p* < 0.001, but not for the 700 ms condition.

As reported earlier, we found a significant main effect of sequence, *F*(1,15) = 16.01, *p* = 0.001, with the congruent sequence showing more consistency overall. The advantage of the congruent sequence was observed primarily in the dual-task conditions with continuous bowing movements at the faster rate (IOI = 700 ms). The four-way interaction with articulation, tempo, and task was significant, *F*(1,15) = 21.59, *p* < 0.001. Post hoc comparisons indicated significant differences across Congruent and Incongruent sequences in the discrete (staccato) dual-task performances at the 700 ms rate but not at the 1,100 ms rate; Fig. 4 shows that variability increased for the Incongruent sequence at the 700 ms rate but remained high across sequence types for the 1,100 ms rate, *t*(15) = 3.42, *p* = 0.004. The same comparison across congruent–incongruent sequences in the continuous (legato) dual-task performances were significant at the 1,100 ms rate but not at the 700 ms rate; Fig. 4 shows that variability increased for the Incongruent sequence at 1,100 ms within the dual-task conditions but remained low across both sequence types in the 700 ms condition, *t*(15) = 4.25, *p* = 0.001.

In contrast to an emergent timing model, an alternate timing model adheres to an independent components approach that assumes that temporal intervals are produced using an internal clock and that clock processes are independent of processes involved in motor response implementation (Wing and Kristofferson 1973a, b). This model might predict that increased variability from single-task to dual-task conditions affected only clock processes and not motor processes. We applied Wing and Kristofferson’s (1973b) model to the cello performances to decompose temporal variability in the tone IOIs associated with the clock component and the motor delay component. Those analyses indicated increased variance from single-task to dual-task conditions for both the clock interval component, *F*(1,15) = 17.01, *p* = 0.001, and the motor delay component, *F*(1,15) = 6.61, *p* = 0.021. These findings are tempered by the fact that the lag-1 autocorrelations, expected to yield negative values, fell in the expected (−0.5,0) range for only 55 % of single-task trials and 68 % of dual-task trials (as well, the model is usually applied to data from repetitive tapping tasks in which there is no additional signal present such as the working memory stimuli). Thus, the independent components model failed to provide a better account of the findings reported here, which are more in line with predictions of emergent timing models.

## Discussion

Cellists’ bowing movements provided a natural task for comparison of working memory and production rate effects on the temporal control of discrete and continuous bowing movements (represented by staccato and legato articulation, respectively). We hypothesized that patterns of mutual interference would occur between the timing task and the working memory task. More specifically, we expected that the temporal control of discrete bowing movements, slow production rates, and producing a sequence with incongruent changes in pitch class and movement direction rely on the same (limited) cognitive resources as the working memory task. Each of these hypotheses proved correct: temporal control suffered as a result of interference from working memory, in each of these conditions. We discuss each effect below.

First, the results showed that cellists exhibited higher variability in the presence of a secondary working memory task: this increase in temporal variability was significant for discrete bowing movements only. This finding suggests that the temporal control of discrete (but not continuous) bowing movements relies on cognitive resources shared with working memory. This is in line with previous research suggesting that the temporal control of discrete movements depends upon an event timing system, which is clock-based involving an explicit representation of time, whereas emergent timing is directly related to the control of movement dynamics (Zelaznik et al. 2002, 2005, 2008). We were also interested in the effect of working memory load on the speeding up/slowing down of the cellists’ performance timing (as indicated by the mean tone IOIs). Mean tone IOIs measures indicated a general tendency to shorten temporal intervals when cognitive load increased, consistent with other empirical studies (Çorlu et al. 2014; Krampe et al. 2010; Rattat 2010). However, some studies contradict this finding, showing no effect (Fortin and Breton 1995; Ogden et al. 2011), or even an opposite effect (Brown 2006; Brown et al. 2013). Studies of the effect of a cognitive load on time judgments and related theoretical models reflect this dichotomy (Block 2010). Basically, attention-based models state that the experience of time duration is shortened when attention is diverted to non-temporal tasks (Studenka and Zelaznik 2011). In contrast, memory-based models—such as Ornstein’s storage-size hypothesis (Torre and Balasubramaniam 2009)—state that the experience of duration is related to the amount of stored information: As the storage increases, the duration experience lengthens. The results of our experiment support the memory-based account, as the produced tone intervals became shorter (cf. duration experience lengthens) in more cognitively demanding (complex) conditions (dual-task conditions, incongruent sequences, and discrete bowing movements).

Second, we found that the temporal variability increased significantly at the slower tempo (IOI = 1,100 ms) when playing the melodies under cognitive load. We found that the working memory task did not affect timing variability at the faster tempo (IOI = 700 ms). The production of sub-second intervals (e.g., IOI = 700 ms) has been shown to depend on an automatic timing system that recruits the motor system, while the production of supra-second intervals (e.g., IOI = 1,100 ms) is more cognitively controlled and depends upon prefrontal and parietal regions (Lewis and Miall 2003). These findings support the idea that the temporal production of sub-second intervals recruits an automatic timing system, as a heightened cognitive load did not affect temporal variability. In line with this, Manning and Schutz (2013) demonstrated that the duration of longer, supra-second intervals can be more reliably estimated by subdividing them into shorter sub-second intervals by means of discrete movements (tapping), in contrast to mental timekeeping. Also, Su and Pöppel (2012) showed that (discrete or continuous) body movements may facilitate the extraction of temporal structures such as the subjective pulse in auditory sequences with sub-second intertone intervals.

Third, cellists’ temporal control was also influenced by the congruence in direction of bowing movements with direction of pitch changes. This was expected, as the incongruent musical sequence required chunking of more elements (DDAD) than the congruent sequence (AD AD), which may have increased the cognitive complexity. A closer inspection of the timing patterns (Fig. 4) reveals that performing the incongruent sequence under cognitive load is particularly hard for staccato articulation at the fast tempo (IOI = 700 ms), and for legato articulation at the slow tempo (IOI = 1,100 ms), compared to performance of the congruent sequence. In addition to working memory constraints, these findings may be explained by differences in the technical demands of bowing a longer chunk of musical tones in the incongruent sequence.

The finding that cellists’ continuous bowing movements can enhance timing under cognitive load is important, because it suggests that cognitive resources that serve to produce regular (supra-second) time intervals may be relieved by motor system functions. This finding can be explained by a dynamical systems approach to motor control and coordination in which temporal regularities are emergent properties of a self-organizing system such as the motor control system (Kelso 1997, 2001). In this view, timing emerges from the control of movement dynamics, rather than being explicitly controlled by a central dedicated clock (Zelaznik et al. 2002, 2005, 2008). Until now, most research on the distinction between event and emergent timing systems has focused on production of sub-second intervals. Our research extends the event-emergent paradigm to supra-second production timing. This was realized by connecting the event-emergent paradigm to the hypothesis, outlined in Lewis and Miall (2003), that the specific control mechanisms recruited for temporal production tasks depend on the duration of the intervals to be timed. The findings reported here suggest that emergent timing control is facilitated when people produce supra-second intervals under cognitive load as an alternative for cognitively controlled, event-based timing.

Previous studies of auditory-motor synchronization have shown that synchronization of continuous movements with an external rhythm leads to more variable asynchronies and decreased error correction responses, compared with synchronization of discrete movements (Elliott et al. 2009; Repp and Steinman 2010; Studenka and Zelaznik 2011; Torre and Balasubramaniam 2009). Despite the decreased synchronization performance, people are capable of maintaining accurate interresponse intervals with continuous movements (Studenka and Zelaznik 2011). Comparisons of movement trajectories that arise in discrete tapping tasks and continuous oscillatory tasks has led researchers to propose that movements serve synchronization goals in discrete tasks via error correction, whereas they provide information to implement directly synchronization in continuous oscillations (Torre and Balasubramaniam 2009). Some studies suggest that smooth, continuous movements increase the central nervous system’s uncertainty about the discrepancy between the timing of the planned and produced movements (Elliott et al. 2009; Hogan and Sternad 2007). It is the continuity of movement, its resistance to change, and consequently the implementation of temporal regularity that form the strengths of emergent timing (Repp and Steinman 2010; Torre and Balasubramaniam 2009). The current study examined timing performance in the continuation phase only, which does not require the coupling of self-generated movements with external events; synchronization during ensemble music performance may change the role of discrete and continuous movements in performance timing.

Both continuous and discrete sources of sensory information are available to cellists during performance, arising from auditory, tactile, and visual feedback. These sensory information streams exhibit particular temporal cues that may function as a “scaffold” in support of temporal control and coordination of body movements (cf. dynamic attending theory Jones and Boltz 1989). Studies of sensorimotor synchronization have shown that the continuity of external rhythms influences temporal coordination. For instance, Varlet et al. (2012) showed that discrete versus continuous stimulus rhythms differentially affect the phase of the synchronized movements: tappers’ movements preceded the onsets of continuous stimuli but lagged the onsets of discrete stimuli. This tendency to anticipate continuous rhythms was also found by Rodger and Craig (2011), who demonstrated smaller synchronization errors when movements were synchronized with discrete sounds, but smaller variability in movement timing when movements were synchronized with continuous sounds. As well, sensorimotor synchronization appears to be influenced by modality: synchronization performance across stimulus rhythms was less variable for auditory presentation than for visual presentation (Zarco et al. 2009). Also, specific interactions may exist between different modalities; when tactile information is minimized, the presence of visual information weakens auditory-motor synchronization (Bravi et al. 2014) and some evidence suggests that visual and auditory rhythms are integrated together in order to improve synchronization performance, irrespective of their continuity (Varlet et al. 2012). In sum, these studies provide evidence that the control processes underlying sensorimotor synchronization differ depending on the nature of the external sensory rhythms.

## Conclusion

The current study investigated the relation between temporal control and working memory, and the roles of movement type (discrete vs. continuous) and tempo (sub-second vs. supra-second tone IOIs) in cellists’ naturalistic performance of musical sequences with bowing movements that were congruent or not with the pitch changes. The findings suggest that the performance of continuous (but not discrete) bowing movements can compensate for disrupting effects of cognitive load on timing production. This effect was greatest at the slower tempo (supra-second IOIs). These findings pinpoint the importance of both movement type and tempo in the role of working memory in fluent tasks like music performance. This research contributes to fundamental knowledge on temporal aspects of human behavior and the neural underpinnings of this behavior, as well as to practical outcomes in fields of music education, motor rehabilitation, and sports, which require the fast and accurate production of rhythmic movement sequences.
